# Asymmetric division of stem cells and its cancer relevance

**DOI:** 10.1186/s13619-024-00188-9

**Published:** 2024-02-27

**Authors:** Shanshan Chao, Huiwen Yan, Pengcheng Bu

**Affiliations:** 1 Key Laboratory of Epigenetic Regulation and Intervention, Chinese Academy of Sciences, Beijing, 100101 China; 2 Key Laboratory of RNA Biology, Institute of Biophysics, Chinese Academy of Sciences, Beijing, 100101 China; 3 College of Life Sciences, University of Chinese Academy of Sciences, Beijing, 100049 China

**Keywords:** Stem cell, Asymmetric division, Cell fate, Cancer

## Abstract

Asymmetric division is a fundamental process for generating cell diversity and maintaining the stem cell population. During asymmetric division, proteins, organelles, and even RNA are distributed unequally between the two daughter cells, determining their distinct cell fates. The mechanisms orchestrating this process are extremely complex. Dysregulation of asymmetric division can potentially trigger cancer progression. Cancer stem cells, in particular, undergo asymmetric division, leading to intra-tumoral heterogeneity, which contributes to treatment refractoriness. In this review, we delve into the cellular and molecular mechanisms that govern asymmetric division and explore its relevance to tumorigenesis.

## Background

Stem cells are characterized by their inherent capacity for self-renewal and the generation of differentiated cells, achieved through two primary strategies: symmetric cell division and asymmetric cell division. Symmetric cell division serves the purpose of either replenishing the stem cell pool or producing terminal differentiated cells. This phenomenon is frequently observed during various processes, such as development, wound healing, and regeneration. In contrast, asymmetric division is employed by stem cells to yield both a new stem cell for self-renewal and a differentiated cell, fostering cellular diversity. This mechanism plays a pivotal role in maintaining the stem cell population and ensuring tissue homeostasis. (Chhabra and Booth 2021; Inaba and Yamashita 2012). Asymmetric division differentially segregates cell fate determinants, including proteins, organelles and RNAs, into the two daughter cells. This intricate process is meticulously orchestrated and controlled by intrinsic and extrinsic factors. (Knoblich 2008; Sunchu and Cabernard 2020; Venkei and Yamashita 2018). A multitude of studies have conclusively demonstrated that the disruption of asymmetric cell division disrupts the delicate equilibrium between self-renewal and differentiation. This disruption can lead to the interruption of differentiation and, in more serious cases, trigger the progression of cancer (Bajaj et al. 2015; Bu et al. 2016; Choi et al. 2020b; Clevers 2005). In this review, we provide a concise overview of the fundamental mechanisms of asymmetric division and discuss how dysregulation can lead to oncogenesis.

## Mechanisms of asymmetric cell division

Asymmetric cell division is a highly coordinated process that depends on both intrinsic and extrinsic fate-determining factors. Current research has predominantly utilized model systems such as *Drosophila*, *Caenorhabditis elegans*, and mammals (de Torres-Jurado et al. 2022; Jankele et al. 2021; Loeffler et al. 2019).

### Intrinsic factors

**Fig. 1:**
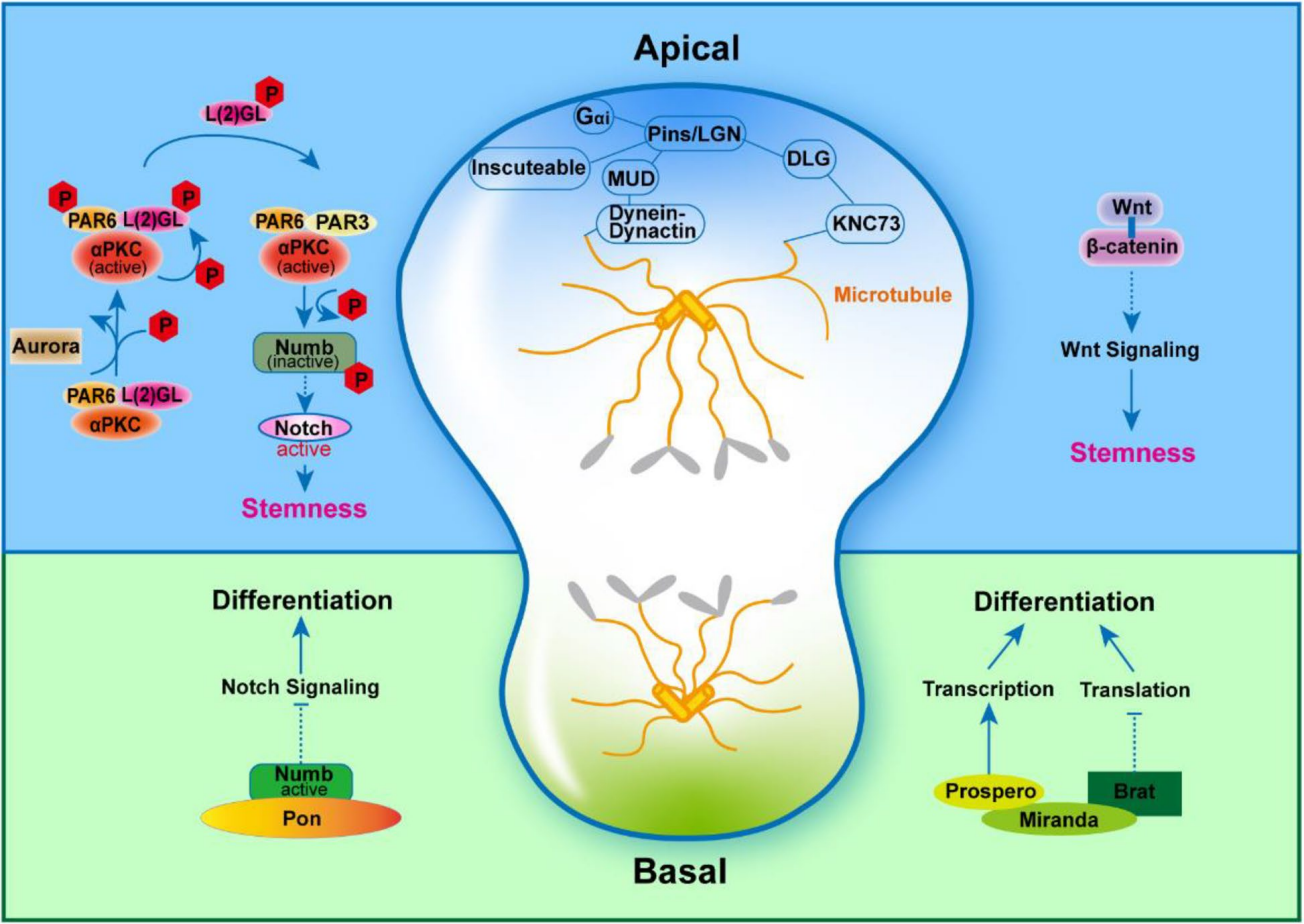
The apical and basal determinants in asymmetric division. On the apical side, aPKC, PAR6 and L(2)GL form a complex that is phosphorylated by Aurora A. Then, aPKC phosphorylates L(2)GL. Phosphorylated L(2)GL is released from the complex and replaced by PAR3. The newly formed complex phosphorylates Numb and leads it releasing from the apical side to the basal side, increasing Numb levels at the basal side and maintainng Notch signaling activity at the apical side. Besides, Wnt signaling is also involved in the stemness maintenance. At the basal side, the accumulation of Numb suppresses the activation of Notch signaling. In addition, the adapter protein Miranda binds to Prospero and Brat at the basal side. After degradation of Miranda, Prospero and Brat are released. Prospero acts as a transcription factor to initiate differentiation. Brat works as a translational repressor to downregulate proteins associated with proliferation. Apical microtubule arrangement is also important during asymmetric division. Inscuteable forms a complex with Pins and Gai and then bind to MUD/DLG/KNC73 complex

Fate-determining protein complexes and spindle assembly are two critical intrinsic factors for asymmetric cell division (Fig. 1). In *Drosophila* neuroblasts (NBs), asymmetric division is regulated by both apical determinants and basal determinants. Apical determinants include protein kinase C (aPKC), partition defective 6 (PAR6), and lethal giant larvae [L(2)GL]. Basal determinants include Miranda, Brat, and Prospero. During the division interphase, aPKC translocates to the apical side and forms a complex with PAR6, a process that leads to the activation of aPKC upon phosphorylation of PAR6. The activated aPKC subsequently reduces its affinity with this complex, resulting in the phosphorylation of Numb. Numb is well-established as a suppressor of Notch signaling. The phosphorylation of Numb can activate Notch signaling, thereby conferring stem cell properties upon the apical daughter cell (Knoblich 2010; Mukherjee et al. 2015). In mammalian stem cells, the small GTP-binding protein CDC42 promotes the accumulation of the aPKC/PAR6/PAR3 complex at the apical side, thereby ensuring the integrity of the apical adheren junction (Gallaud et al. 2017; Mukherjee et al. 2015). Conversely, at the basal side, the adapter protein Miranda in the basal determinants complex undergoes degradation. This degradation leads to the release of Prospero (a transcription factor, known as PROX1 in vertebrates), initiating a transcription program that promotes cellular differentiation. Recently, some studies revealed that protein condensates are extensively involved in regulating the asymmetric cell division process such as the polarized distribution and function of apical and basal cell fate determinants. Par complex, Numb and Pon complex as well as Prospero condensates formation could all mediated by phase separation (Liu et al. 2020a, 2020b; Shan et al. 2018).

During metaphase and telophase, spindle assembly is crucial to support asymmetric cell division. The cytoskeletal adapter protein Inscueable relocates to the apical side and binds to the Dynein-Dynactin complex localized at the ends of microtubules, effectively locking spindle orientation (di Pietro et al. 2016). Simultaneously, on the same side, microtubule-bound kinesin Khc73 forms a complex with disc large (Dlg) and Pins. This larger complex links with the apical cytoskeleton through Inscuteable, ensuring precise spindle orientation (Bajaj et al. 2015; Culurgioni et al. 2018; Siegrist and Doe 2005). Beyond these intrinsic cellular mechanisms, external cues also play a role in guiding spindle orientation, highlighting the coordination of this intricate process (Lechler and Mapelli 2021).

### Extrinsic factors

**Fig. 2:**
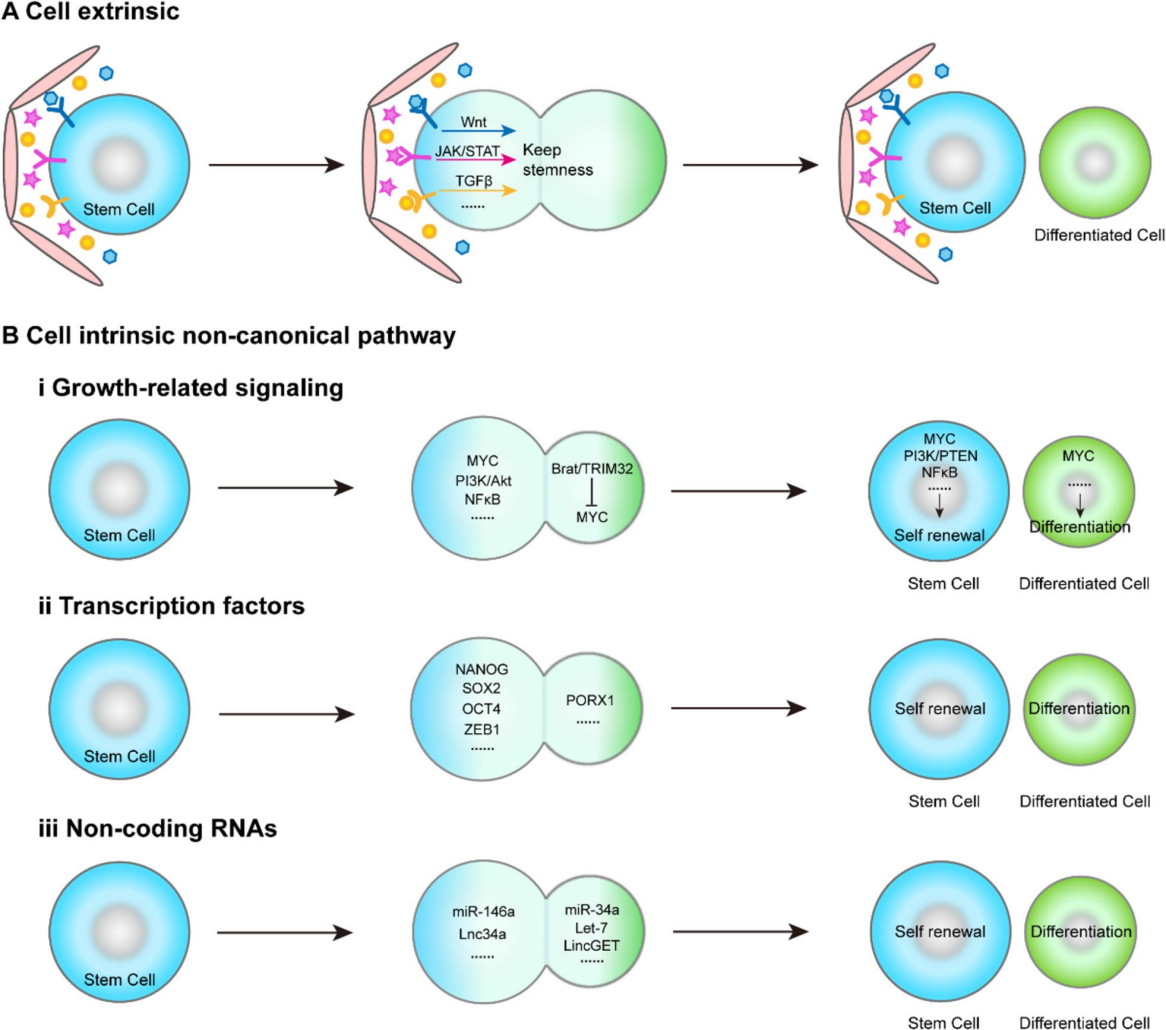
Cell extrinsic and intrinsic cues during asymmetric division. **A** Extracellular microenvironment (local niche) provides cell extrinsic cues to induce asymmetric stem cell division. **B** Cell intrinsic non-canonical pathway is involved in regulating asymmetric stem cell division. Some growth-signaling pathways are involved in regulating asymmetric division of stem cells, such as MYC. On the other side, Brat (*Drosophila*) or TRIM32 (mammalian) ubiquitinates c-MYC leading to cell differentiation (i). Some self-renewal or differentiation transcription factors are accumulated on the side of stem cell or differentiated cell to regulate asymmetric division (ii). microRNAs regulate asymmetric division of stem cells. miR-146a and Lnc34a accumulating in the stem cell side drives stem renewal, whereas miR-34a, Let-7 and LincGET accumulating in the other side drives cell differentiation (iii)

Extracellular microenvironment surrounding stem cells is called stem cell niche, which also influences asymmetric cell division. Stem cell niche usually provide outside signals (such as ligands) to regulate downstream transcription activity and determination of cell fate in stem cells (Fig. 2A). *Drosophila* germline stem cells (GSCs) offer a classic illustration of how extrinsic factors influence asymmetric division (Kahney et al. 2019; Venkei and Yamashita 2018). In the *Drosophila* ovary and testis, the stem cell niches are referred to as the "cap" and the "hub," respectively. Specifically, hub cells secrete critical self-renewal ligands such as Unpaired (Upd, a cytokine homologue) and Decapentaplegic (Dpp)/Glass bottom boat (Gbb), while cap cells secrete the ligand Dpp along with terminal filament cells. Consequently, when stem cells attach to this niche, their stemness is preserved; conversely, being situated farther away triggers the differentiation process (Herrera and Bach 2019; Venkei and Yamashita 2018). In the context of *C. elegans* and *Drosophila* embryonic stem cells, an intriguing phenomenon emerges: the daughter cell in close proximity to Wnt signaling sustains its stemness, whereas the other daughter cell, situated farther from Wnt, initiates the differentiation process (Habib et al. 2013). Furthermore, analogous niche phenomena have been identified in mammalian systems. The niche provides polarity and localized signals that determine the fate of mammalian stem cells progeny through either symmetric or asymmetric cell divisions. In much reported studies, epidermal stem cell, muscle stem cell, intestinal stem cells (Lgr5 + stem cells), etc., are classic types of stem cells regulated by their ecological niche during growth and development. These intricate processes involve a range of cell growth factors and signaling pathways associated with stemness, including TGFβ, Wnt, and sonic hedgehog pathway (Espinoza et al. 2013; Fuchs and Blau 2020; Mullen and Wrana 2017; Santoro et al. 2016). It is noteworthy that the asymmetric division of stem cells is regulated by a combination of intrinsic and extrinsic factors, giving rise to distinct growth outcomes.

### Other noncanonical signaling pathway

In addition to the factors mentioned above, some noncanonical signaling pathways may influence asymmetric cell division directly or indirectly. For example, MYC (C-Myc, N-Myc, L-Myc) signaling pathway is a key regulator of cell growth, proliferation and development, and plays an important role in stem cell maintenance (Dang 2012). Previous study demonstrates that dMyc (*Drosophila* Myc) is expressed in stem cells but not in differentiated cells in *Drosophila* NBs and GSCs (Quinn et al. 2013). This is because Brat inhibits C-Myc expression during asymmetric division in NBs and in GSCs (Harris et al. 2011). In mouse models, TRIM32 (*Drosophila* Brat) is an ubiquitin ligase for C-Myc, which lead to C-Myc degradation in neural progenitors in neocortex (Fig. 2B) (Knoblich 2010).

Certain transcription factors, including Nanog, Sox2, and Oct4, accumulate asymmetrically in stem cells and play a role in determining cell fate (Goolam et al. 2016; Habib et al. 2013). In *Drosophila*, Hippo signaling pathway is participate in modulating asymmetric cell division (Keder et al. 2015).

Besides this, increasing studies supports the critical role of microRNAs in asymmetric division. In our prior study, we identified miR-34a as a pivotal determinant of cell fate in this process. Our findings revealed that miR-34a predominantly localizes in differentiated daughter cells, where it represses Notch signaling (Bu et al. 2013a). Similarly, Let-7 also localizes in differentiated daughter cells (Esquela-Kerscher and Slack 2006). While miR-146a localizes in stem cell and drives it self-renewal (Hwang et al. 2014; Lerner and Petritsch 2014). A recent study shows that LincGET is transiently and asymmetrically expressed in the nucleus of late two-cell blastomere stage of mouse embryos (Fig. 2B) (Wang et al. 2018).

## Asymmetric segregation of cellular components

### Asymmetric inheritance of cellular organelles

In addition to molecular determinants (such as RNAs and proteins), cell organelles included centrosomes, mitochondria, lysosomes and endosomes have been described to segregate asymmetrically.

#### Centrosomes

**Fig. 3:**
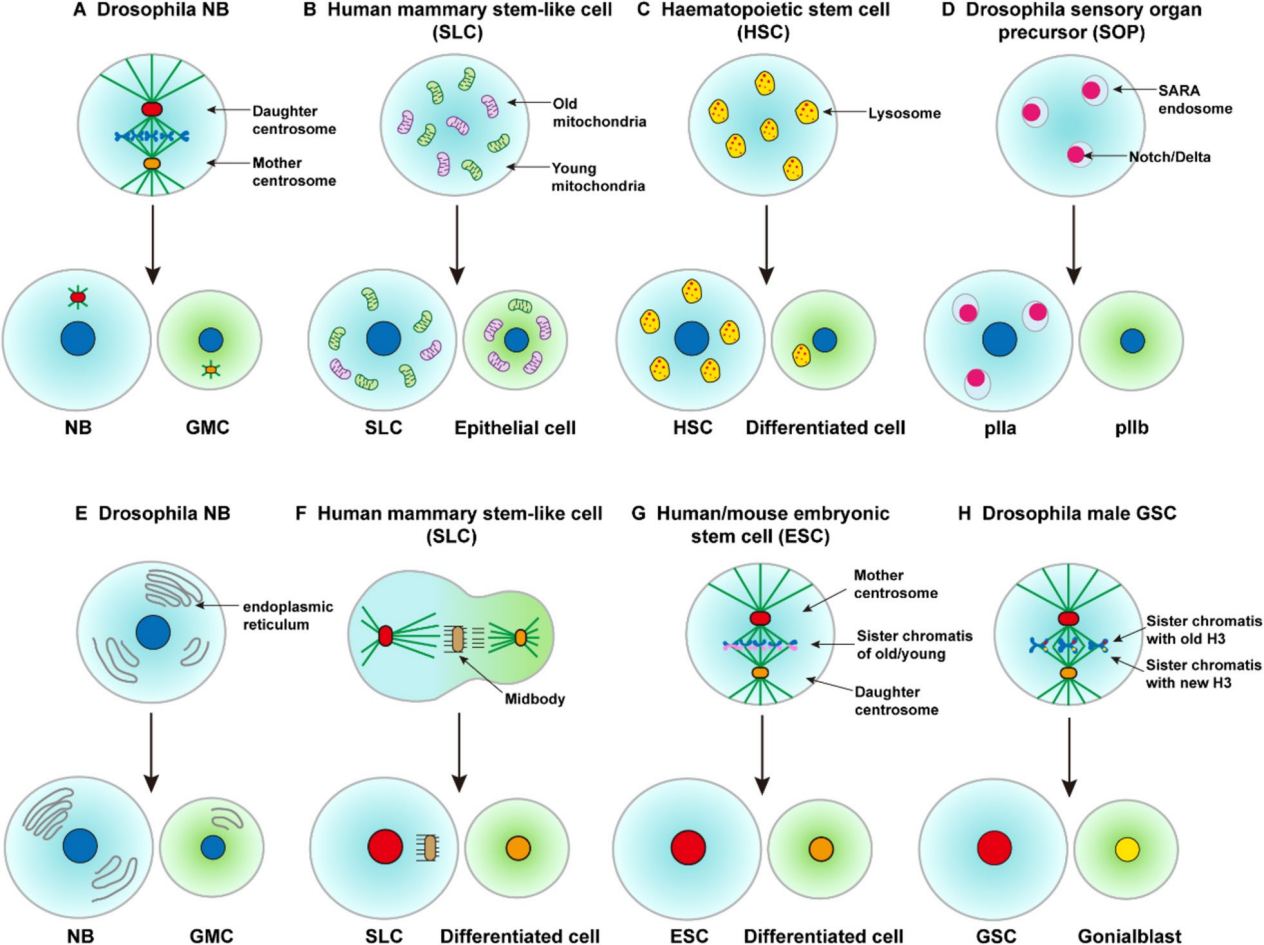
Different partition of cellular components during asymmetric division. The organelles, including **A** the centrosome, **B** the mitochondria, **C** the lysosome, **D** the endosome, **E** the endoplasmic reticulum and other cellular components such as **F** the midbody, **G** the sister chromatids, **H** the histones are asymmetrically inherited by two daughter cells

Centrosome duplicate to generate two daughter centrosomes. Having two centrosomes with different size is crucial for ensuring proper spindle orientation during asymmetric division of *Drosophila* male GSCs and NBs (Rebollo et al. 2007; Yamashita et al. 2003). For instance, in *Drosophila* male GSCs and mouse radial glial progenitors (RG), the mother centrosome is preferentially inherited (Wang et al. 2009; Yamashita et al. 2007); Conversely, in *Drosophila* NBs and female GSCs, the daughter centrosome is preferentially segregated into the stem cells (Fig. 3A) (Conduit and Raff 2010; Salzmann et al. 2014). Recently, Royall et al. found asymmetric inheritance of centrosomes is a mechanism which to maintain self-renewal properties and to ensure proper neurogenesis in human neural progenitor cells (Royall et al. 2023).

#### Mitochondria

Mitochondria are crucial organelles for supplying cellular energy, and their accurate segregation plays a critical role in determining cell fate. The asymmetric allocation of mitochondria is indispensable for maintaining stemness properties. The daughter cell inheriting stem cell traits typically possesses fewer aged mitochondria. In contrast, the other daughter cell, with a higher proportion of aged mitochondria, is more inclined towards differentiation (Fig. 3B). This process relies on both mitochondrial fission and the machinery for quality control of mitochondria (Bockler et al. 2017; Katajisto et al. 2015).

#### Lysosome

A recent study demonstrates that lysosomes are asymmetrically inherited in human neural stem cells (NSCs). These lysosomes house Notch receptors, and the acidic environment within them can activate Notch signaling. Consequently, lysosomes serve as a signaling hub, leading to differential Notch signaling activity between the daughter cells during asymmetric division (Bohl et al. 2022).

Lysosomes and active mitochondria are asymmetrically inherited in human blood stem cells and that their inheritance is a coordinated, nonrandom process (Fig. 3C). Furthermore, multiple additional organelles, including autophagosomes, mitophagosomes, autolysosomes, show preferential asymmetric cosegregation with lysosomes. Importantly, asymmetric lysosomal inheritance predicts future asymmetric daughter cell-cycle length, differentiation, and stem cell marker expression, whereas asymmetric inheritance of active mitochondria correlates with daughter metabolic activity. Hence, human hematopoietic stem cell fates are regulated by asymmetric cell division, with both mechanistic evolutionary conservation (Filippi 2022; Loeffler et al. 2022).

#### Endosomes

Asymmetric segregation of SARA (Smad anchor for receptor activation) endosomes, which carry both Notch and its ligand Delta, has been observed in *Drosophila* sensory organ precursor cells (SOPs) during asymmetric division. In this process, SARA endosomes preferentially segregate into pIIa, leading to enhanced Notch activation and reduced stemness in pIIb (Fig. 3D) (Coumailleau et al. 2009). This biased distribution of SARA endosomes has also been documented in colon stem cells and the central nervous system of *Drosophila* (Coumailleau et al. 2009; Montagne and Gonzalez-Gaitan 2014), as well as in neural precursors of the spinal cord in zebrafish (Kressmann et al. 2015). Consequently, the SARA endosome system plays a crucial role in determining the fate of daughter cells during asymmetric division of stem cells.

#### Endoplasmic reticulum

The endoplasmic reticulum (ER) is the largest membrane-bound organelle, essential for lipid and protein biosynthesis. Reports of asymmetric segregation of ER have emerged in *Drosophila* larval neuroblasts and early *C.elegans* embryos (Fig. 3E). Studies demonstrate that the precise segregation of ER relies on the process of asymmetric nucleation of astral microtubules, a mechanism conserved across various animal species. Consequently, the daughter stem cell resulting from asymmetric division exhibits a higher concentration of ER compared to the differentiating daughter cell (Smyth et al. 2015).

#### Midbody

In mammalian cell cultures, midbody remnants seem to be inherited asymmetrically. They are always retained by the cell with the mother centrosome (Fig. 3F). Midbody remnants were found to associate with several stem cell compartments in vivo like basal layers of mouse testes seminiferous tubules, ventricular progenitor cells in the mouse brain, mouse skeletal muscle progenitors, and the bulge of human hair follicles. Midbodies are accumulated in pluripotent stem cells when cells are dedifferentiated. Remarkably, in stem cells, midbody accumulation seems to positively affect reprogramming efficiency, while for cancer cells, midbody accumulation results in enhanced tumorigenicity (Dionne et al. 2015).

### Sister chromatids

In mouse satellite cells (muscle stem cells) and human/mouse embryonic stem cells, biased sister chromatid segregation is a common occurrence. During asymmetric division, old DNA strands tend to segregate preferentially to the less-differentiated cells (Fig. 3G) (Conboy et al. 2007; Rocheteau et al. 2012). “Immortal Strand Hypothesis” posits that long-lived cells like stem cells may employ this mechanism to avoid the accumulation of mutations during DNA replication (Cairns 1975, 2006; Lark 1966; Potten et al. 1978; Rando 2007). Cells that preserve “immortal strands” will avoid the accumulation of replication error. It seems that the combination of immortal strands and the choice of death rather than errorprone repair makes stem cell systems resistant to short exposures to DNA-damaging agents, because the stem cell accumulates few if any errors, and any errors made by the daughters are destined to be discarded. The histone H2A variant H2A.Z shows specific immunodetection on immortal DNA chromosomes. The unique H2A.Z detection pattern is likely to be an important feature of the molecular mechanisms responsible for nonrandom segregation by asymmetric division (Huh and Sherley 2011). Additionally, in male GSCs, the sister chromatids of X and Y chromosomes exhibit a striking bias in segregation, a process that heavily relies on the centrosome (Yadlapalli and Yamashita 2013).

### Histone

An increasing number of studies have revealed that the asymmetric inheritance of histones also takes place during the asymmetric division of stem cells. In *Drosophila* male GSCs, the older H3 and H4 histones preferentially segregate to the self-renewed GSC, whereas newly synthesized H3 exhibits a bias towards accumulation in the differentiating daughter cell (Fig. 3H). This biased segregation of histones is mediated by distinguishing the phosphorylation at threonine 3 of H3 (H3T3P) between pre-existing and newly synthesized H (Wooten et al. 2019; Xie et al. 2015, 2017). The asymmetric partition of H3 and H4 histones also has been found in *Drosophila* intestinal stem cells (Zion et al. 2023). In contrast, some studies argue that H2A and H2B histones are inherited symmetrically during the asymmetric division of GSCs (Wooten et al. 2020, 2019).

## Asymmetric division in cancer

Asymmetric division of stem cells plays a crucial role during embryogenesis, development, and tissue regeneration. This process involves cell polarity factors, cell fate determinants, and the spindle apparatus in the regulation of stem cell division. Dysregulation or mutation of these factors may lead to a shift from asymmetric to symmetric cell division, and in some cases, even trigger initiation and drug resistance.

## Asymmetric division in cancer initiation

Disruption of asymmetric division in tumor cells is a significant contributor to cancer initiation. In leukemia, dysregulated asymmetric division of Leukemia Stem Cells (LSCs) can lead to the progression of hematologic malignancies. For instance, the mutation of NUP98-HOXA9 has been shown to shift asymmetric divisions towards symmetric renewal divisions. This contributes to increased self-renewal, differentiation arrest, and progression (Bajaj et al. 2015; Wu et al. 2007). Previous studies have also highlighted changes in specific factors during the progression of Chronic Myeloid Leukemia (CML). Notably, Numb is downregulated, while the repressor of Numb, Msi (an RNA-binding protein), is significantly upregulated. Deletion of Msi2 restores Numb expression and inhibits the development of CML in a mouse leukemia mode (Ito et al. 2010). Similarly, Msi2 is highly expressed in Acute Myeloid Leukemia (AML) cell lines, indicating a positive correlation with high-grade hematologic malignancies (Kharas et al. 2010).

In colon cancer, miR-34a, identified as a p53 target, typically acts as a tumor suppressor. It exerts its influence by binding to the 3’UTR of mRNA. Deficiency of miR-34a disrupts the balance between self-renewal and differentiation, ultimately enhancing symmetric division of cancer stem cells in colorectal cancer (Bu et al. 2013a). Remarkably, long non-coding RNA, Lnc34a, is enriched in colon cancer stem cells and initiates asymmetric division by directly targeting the microRNA miR-34a to cause its spatial imbalance (Wang et al. 2016). In other studies, miR-146a was found to activate WNT signaling and induce a switch from asymmetric to symmetrical division by targeting Numb in spheroid-derived colorectal cancer stem cells (Hwang et al. 2014). In breast cancer, The Myc signaling pathway is essential for stem cell maintenance and is predominantly expressed in stem cells, not in differentiated cells, during asymmetric cell division. Myc plays a pivotal role in tumor growth through processes like gene amplification and translocation (Lourenco et al. 2021; Zheng et al. 2008). In a guanine nucleotide-dependent mechanism, the p53 antioncogene can induce exponentially dividing cells to switch to an asymmetric stem cell growth pattern. Sherley et al. develops engineered cultured cells that exhibit asymmetric self-renewal and immortal DNA strand cosegregation. It suggests that the observed high frequency of p53 mutations in human cancers reflects a critical function in the regulation of somatic renewal growth (Rambhatla et al. 2005).

## Asymmetric division in cancer drug resistance

Recent study show that asymmetric division of colorectal cancer stem-like cells is critical for early intratumor heterogeneity establishment. Targeting the asymmetric division of cancer stem-like cells can change tumor heterogeneity and thus contribute to the therapy of colorectal cancer (Chao et al. 2023). Consistent with this conclusion, asymmetric divisions contribute to the generation of intratumoral heterogeneity has also been identified in triple-negative basal-like breast cancers (Granit et al. 2018). Polyploid giant cancer cells (PGCCs) are key contributors to the cellular heterogeneity and play a fundamental role in regulating stemness, and chemoresistance in cancers. Polyploidy disturbs the overall transcription level to upregulate genes promoting cell growth and chemoresistance. Asymmetric cell division of giant cancer cells by meiosis-like depolyploidization is proposed to explain the unexpected life cycle of these cells. Cell cycle-related proteins, such as cyclin E and cyclin D1, are important in regulating the asymmetric division of PGCC. Expression levels of cyclin E and cyclin D1 were much higher in PGCCs compared with that in diploid cancer cells. PGCC formation is regulated by recompartmentalization of cell cycle regulatory proteins normally involved in the regulation of asymmetric division (Zhang et al. 2014; Zhou et al. 2022).

## Conclusions

Asymmetric division of stem cells is an accurately regulated process. It balances self-renewal and differentiation in cancer cells through generating two unequal daughter cells. These two daughter cells are different in distinct fate, function, and size. One daughter retains stem cell properties like itself while the other enters into a differentiation program. It is a convenient way to maintain the stem cell populations and enrich cell-type diversification (Bajaj et al. 2015; Sunchu and Cabernard 2020). Thus, asymmetric stem division is a vital process for development and tissue homeostasis.

As mentioned above, asymmetric cell division is achieved through highly coordinated intra- and extracellular biological processes. These crucial events encompass the localization of fate-determining proteins in apical and basal regions, the asymmetric assembly of spindles and microtubules, as well as the influence of microenvironmental extrinsic factors and other signaling networks (Mukherjee et al. 2015). In mammals, these processes are even more complex, involving a greater number of factors and signaling pathways (Sunchu and Cabernard 2020; Venkei and Yamashita 2018). Normally, stem cells dynamically switch between asymmetric and symmetric division to maintain homeostasis. However, when asymmetric division is dysregulated, there arises a heightened risk of cancer (Bajaj et al. 2015; Choi et al. 2020a; Li et al. 2022).

**Fig. 4:**
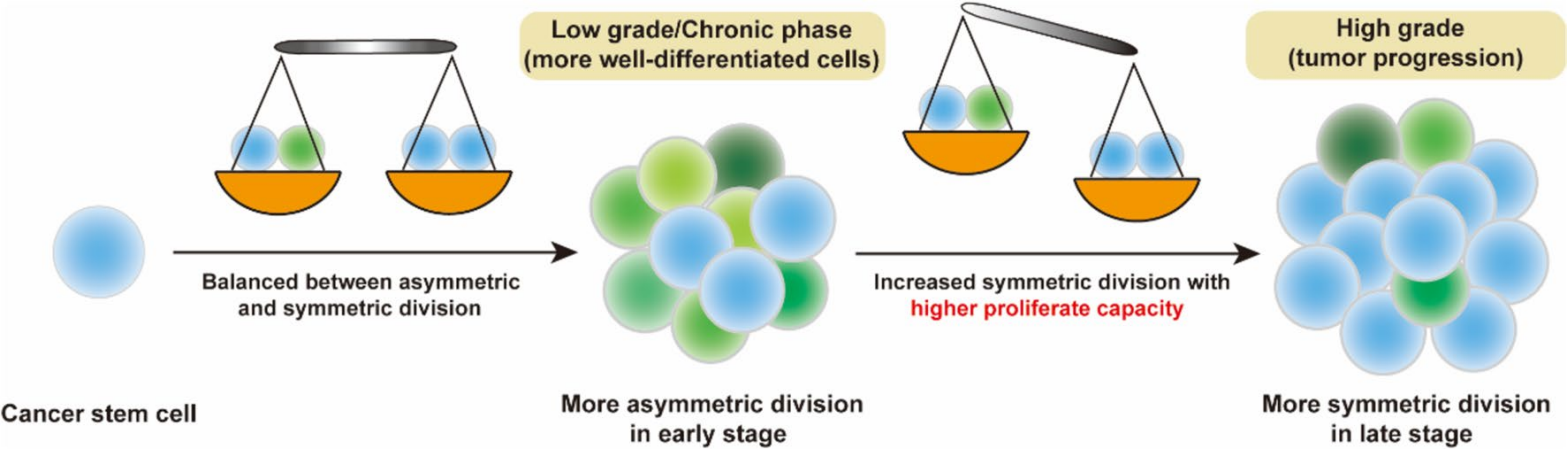
Asymmetric or symmetric division in tumor development. Asymmetric division of cancer stem cells happens frequently in early stage of cancer, which not only maintains the pool of cancer stem cells, but also creates tumor heterogeneity (left). Tumors at this stage are usually well differentiated. However, the balance between asymmetric and symmetric division is broken in late stage of cancer. Increased symmetric division of cancer stem cells enables tumor to have higher proliferative capacity thus contributes to tumor progression (right). Tumors at this stage are usually poorly differentiated

Numerous studies have demonstrated that disruptions in asymmetric cell division can lead to enhanced stem cell self-renewal, resulting in overgrowth and triggering tumorigenesis and cancer progression (Fig. 4). Aberrant expression of cell fate determinants, such as aPKC, L(2)GL, PROX, Numb, DLG, and others, is associated with tumors. Additionally, microRNAs associated with self-renewal and differentiation play a relevant role in cancer. They generally sustain activation of the Notch/Wnt signaling pathway or suppress the expression of differentiation-related transcription factors (Bu et al. 2013a; Hwang et al. 2014; Mukherjee et al. 2015). Furthermore, defects in AKT, TP53, and EGFR signaling can disrupt the balance between asymmetry and symmetry, leading to neoplastic transformation (Bu et al. 2013b; Mukherjee et al. 2015). Studies suggest that asymmetric division is negatively correlated with proliferative capacity. More symmetric renewal divisions result in a more undifferentiated and malignant state (Bajaj et al. 2015; Lytle et al. 2018). Therefore, aberrantly shifting from asymmetric division to symmetric division can contribute to cancer progression (Fig. 4).

Understanding the mechanisms of asymmetric division could not only help us identify new targets to suppress tumor but also raise the possibility that transferring symmetrical divisions to asymmetric divisions may be a new therapy strategy for patients in late stage.

## Data Availability

Not applicable.

## Abbreviations

NBs: Neuroblasts
aPKC: Protein kinase C
PAR6: Partition defective 6
L(2)GL: Lethal giant larvae
Dlg: Disc large
GSCs: Germline stem cells
Upd: Unpaired
Dpp: Decapentaplegic
Gbb: Glass bottom boat
RG: Radial glial progenitors
SARA: Smad anchor for receptor activation
SOPs: Sensory organ precursor cells
ER: Endoplasmic reticulum
LSCs: Leukemia Stem Cells
CML: Chronic Myeloid Leukemia
AML: Myeloid Leukemia
PGCCs: Polyploid giant cancer cells
